# Regulation of LL-37 in Bone and Periodontium Regeneration

**DOI:** 10.3390/life12101533

**Published:** 2022-09-30

**Authors:** Zahra Chinipardaz, Jessica M. Zhong, Shuying Yang

**Affiliations:** 1 Department of Basic and Translation Sciences, University of Pennsylvania, 240 South 40th Street, Levy 437, Philadelphia, PA 19104, USA; 2 Department of Periodontics, School of Dental Medicine, University of Pennsylvania, Philadelphia, PA 19104, USA; 3 Center for Innovation & Precision Dentistry, School of Dental Medicine, School of Engineering and Applied Sciences, University of Pennsylvania, Philadelphia, PA 19104, USA; 4 The Penn Center for Musculoskeletal Disorders, School of Medicine, University of Pennsylvania, Philadelphia, PA 19104, USA

**Keywords:** antimicrobial peptide, bone, LL-37, oral cavity, periodontium, regeneration

## Abstract

The goal of regenerative therapy is to restore the structure and function of the lost tissues in the fields of medicine and dentistry. However, there are some challenges in regeneration therapy such as the delivery of oxygen and nutrition, and the risk of infection in conditions such as periodontitis, osteomyelitis, etc. Leucine leucine-37 (LL-37) is a 37-residue, amphipathic, and helical peptide found only in humans and is expressed throughout the body. It has been shown to induce neovascularization and vascular endothelial growth factor (VEGF) expression. LL-37 also stimulates the migration and differentiation of mesenchymal stem cells (MSCs). Recent studies have shown that LL-37 plays an important role in the innate defense system through the elimination of pathogenic microbes and the modulation of the host immune response. LL-37 also manifests other functions such as promoting wound healing, angiogenesis, cell differentiation, and modulating apoptosis. This review summarizes the current studies on the structure, expression, and function of LL-37 and highlights the contributions of LL-37 to oral cavity, periodontium, and bone regeneration.

## 1. Introduction

The innate immune system is the first line of defense against microbes as well as in the initiation of inflammatory responses [1]. When tissues first contact pathogenic microorganisms, antimicrobial peptides (AMPs) are released from precursor proteins [2], which have an important role in initiating the mechanism of host defense [3].

AMPs are evolutionarily conserved molecules and possess a broad antimicrobial spectrum [4,5]. Defensins and cathelicidins are the most prominent family members of AMPs. Mammalian defensins are cationic non-glycosylated peptides with arginine as the primary cationic residue [3,6], which consists of a β-sheet structure and three disulfide bonds. In humans, two classes of defensins can be found: α-defensins and β-defensins based on the difference in the connecting patterns of the three disulfide bonds and the spacing of cysteine [7,8].

The cathelicidin family is characterized by the ‘cathelin’ domain (a conserved N-terminal sequence) and a highly variable C-terminal sequence, which corresponds to the antimicrobial peptide. LL-37 is the only cathelicidin expressed in humans [9]. Recently, the focus has been extended from the antimicrobial functions of LL-37 to other functions of LL-37 including regeneration. One of the essential components in regeneration therapy is growth factors; however, most of the growth factors play roles in promoting regeneration instead of anti-microbia or inflammation. Another critical factor of tissue engineering is neovascularization, which is imperative to supply sufficient oxygen and nutrients to the cells [10]. Provided that LL-37 has broad antimicrobial and angiogenesis effects [11,12,13,14], it may be considered as a promising therapeutic target for regeneration. In this review, we intend to briefly summarize the anti-microbial effect of LL-37 in the oral cavity and then provide the most recent and relevant insights regarding its contribution in periodontium and bone regeneration.

### 1.1. Cathelicidin

The cathelicidins are synthesized as preproproteins [15]. The cathilicidin family contains a highly conserved cathelin domain [15], which is flanked by a signal peptide domain (approximately 30 residues long) on its N-terminus [9]. Once the N-terminus is removed when the cathelicidin accesses the storage granules, the cathelicidin is then called the ‘proprotein’—consisting of the cathelin domain and the C-terminal domain—which is represented in storage form. The C-terminal antibacterial peptides are activated when they split from the proprotein by serine proteases, which form the active AMPs [16,17,18,19]. There is a large variation in the amino acid sequence and structure of the C-terminal ranging from proline- and arginine-rich sequences to sequences forming amphipathic α-helices [19].

Cathelicidins were first discovered by Romeo et al. They isolated a disulfide-containing cyclic dodecapeptide from bovine neutrophil lysates [20] followed by the purification of two additional neutrophil antimicrobial peptides—bactenecins—, Bac5 and Bac7, and found that they had antimicrobial activity [21]. Zanetti et al. cloned the DNA of Bac5 from bovine bone marrow mRNA and discovered remarkable similarity to the cathelicidins inhibitor of the cysteine proteinase cathepsin L in pigs [22,23]. In 1995, Zanetti et al. proposed the ‘cathelicidins’ term based on the presence of a common cathelin-like domain in the specific AMPs’ precursors and it is used to denote molecules that contain a cathelin-like sequence and a cationic antimicrobial domain [15]. Cathelicidins have been discovered in many other mammalian species and even non-mammalian species. More information on the different types of species which express cathelicidins was reviewed by Weistroffer et al. [24]. It is very interesting to note that two cathelicidins have been discovered in hagfish which is the oldest living member of jawless craniates and deficient in essential components of adaptive immunity [25,26].

Most species have multiple cathelicidin genes with different functions; however, humans, mice, and rats express only one cathelicidin gene with many functions, denoted as hCAP18 (human cationic antimicrobial protein)/LL-37, mCRAMP (cathelicidin-related antimicrobial peptide), and rCRAMP [27], respectively.

hCAP18/LL-37 has been largely studied for its antibacterial (both Gram-positive and Gram-negative) [11,12,13], antiviral [12,28,29,30], antifungal [31,32], and antiparasitic [33] effects. Cathelicidin is also capable of inhibiting protease cathepsin-L activity [34]. Recently, increasing evidence has shown that it possesses other functions such as regeneration which will be discussed in this review.

### 1.2. LL-37

#### 1.2.1. Structure of LL-37

**Figure 1:**
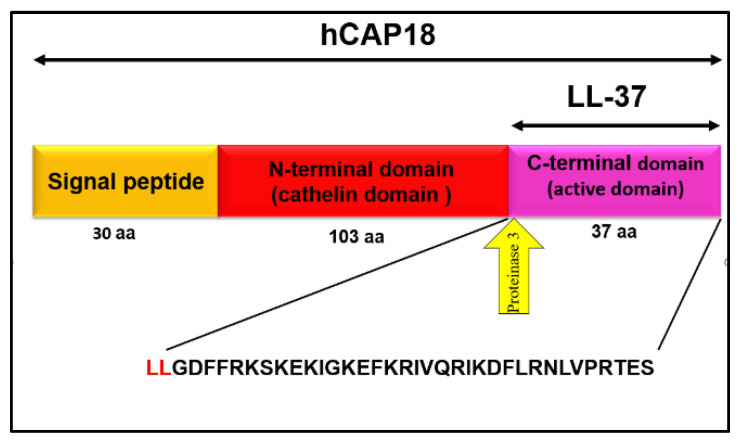
Schematic representation of hCAP18 and LL-37.

LL-37 is the sole cathelicidin family member in humans, which was identified in 1995 and first named hCAP18 due to its close similarities to the rabbit’s cationic antimicrobial and it has a molecular weight of 18 kDa [35]. hCAP-18 consists of an N-terminal signal sequence (103 amino acid residues), a well conserved cathelin-like domain, and a C-terminal antimicrobial peptide domain composed of 37 amino acids starting with a pair of leucines (LLGDFFRKSKEKIGKEFKRIVQRIKDFLRNLVPRTES) [27], so it is also named LL-37. The peptide is derived by the extracellular cleavage of proteinase 3 from the C-terminal end of hCAP18 [19]. The schematic structure of the LL-37 is shown in Figure 1.

LL-37 is located in chromosome 3 with four exons. Only exon 4 codes the antimicrobial part and exons 1 to 3 code for the signal and the cathelin domains [36]. LL-37 forms a random coil in pure water; however, it shapes a cationic amphipathic α-helical structure in the presence of membranes or in solutions with salts [37]. The four aromatic phenylalanine rings (Phe-5, Phe-6, Phe-17, and Phe-27) chains form the hydrophobic surface of LL-37, which is surrounded by predominately positively charged residues for the recognition of negatively charged structures, such as bacterial cell walls, and LPS [38].

The N-terminal region of LL-37 has been implicated in chemotaxis, hemolytic activity, proteolytic resistance, and peptide oligomerization, but is less responsible for antibacterial activity [39,40,41]. The C-terminal region of LL-37 is important for the antimicrobial, anticancer, and antiviral effects [42].

#### 1.2.2. Expression and Function

The LL-37 expression pattern has been summarized by Durr et al. [43]. It is known that LL-37 expresses in a variety of cells such as epithelial cells [44,45,46,47,48,49], immune cells [50,51,52,53,54,55], ocular surface [12] and eccrine glands [11], mesenchymal stem cells [56], and bone marrow stroma [57]. Moreover, LL-37 has been detected in body fluids such as breast milk [58,59], sweat [60], saliva [61,62], and semen [63].

LL-37 exerts its antimicrobial activity through pore formation in Gram-positive and Gram-negative bacteria and can neutralize LPS [64,65,66,67]. In addition to its antimicrobial properties, LL-37 affects other cellular functions, such as phagocytosis [68], cell differentiation [46], and apoptosis [69,70,71,72,73]. Interestingly, the impact of LL-37 on apoptosis depends on the cell type. For example, LL-37 has an inhibitory effect on the apoptosis of keratinocytes [70] and dermal fibroblasts [66]. However, LL-37 induces apoptosis in vascular smooth muscle cells [74], periodontal ligament cells [75], neutrophils [76], airway epithelium [73], and T cells [77]. Moreover, LL-37 expresses immunomodulatory effects and plays a crucial role in innate immunity [78,79,80]. For instance, LL-37 downregulates interleukin (IL)-10 expression and upregulates IL-12p40 expression leading to a pro-inflammatory response during monocyte to macrophage differentiation [81]. LL-37 promotes inflammatory cytokine production via the regulation of IL-1β signaling [82]. Moreover, LL-37 chemoattracts mast cells [83], monocytes, T lymphocytes, and neutrophils [84] and induces mast cell degranulation [85]. Anti-inflammatory effects of LL-37 include antagonistic action on IFNγ, TNF-α, IL-4, and IL-12 responses in different cell types [69,82,86]. LL-37 lowers inflammatory cytokine production in the presence of LPS via the interruption of the toll-like receptor (TLR)-4 receptor complex function in macrophages and dendritic cells [87,88]. Thus, the microenvironment may modulate both the pro- and anti-inflammatory functions of LL-37 [80].

Besides these functions, LL-37 can also regulate angiogenesis [14,89,90,91] and wound healing [92,93,94,95]. Fibroblasts synthesize the extracellular matrix, which is indispensable for wound healing and tissue repair. A study by Krasnodembskaya et al. showed that the antimicrobial property of human MSCs is partially mediated by the secretion of LL-37 [96]. Recently, LL-37 has been applied successfully in tissue regeneration due to its angiogenesis effect and recruitment of MSCs [89,97].

## 2. Effects of LL-37 on Bone Cells

### 2.1. Osteoclasts

It is well known that bone tissue continuously remodels throughout life in which osteoclasts (bone-resorbing cells) resorb the old bone followed by bone-forming cells (osteoblasts) which form bone [98]. Osteoclasts are multinucleated cells differentiated from monocyte/macrophage-lineage precursors with the stimulation of two cytokines: (1) macrophage colony-stimulating factor (M-CSF) and (2) receptor activator of nuclear factor kappa-B ligand (RANKL) [99,100].

Supanchart et al. cultured human peripheral blood mononuclear cells (PBMCs) in an osteoclastogenic medium with LL-37 and found that monocytes do not differentiate into osteoclasts in the presence of LL-37. Interestingly, they further found that monocytes maintained their viability at low doses of LL-37 (2–10 µM); however, most of monocytes die at high doses of LL-37 (20 µM and 30 µM). Additionally, LL-37 simulates M-CSF production and maintains monocyte viability even without the exogenous addition of M-CSF [101]. Consistently, Takayanagi, H et al. found that LL-37 inhibits NFAT2 (Nuclear factor of activated T cell) nuclear translocation, which is essential for osteoclastogenesis [102]. In LL-37 treated PBMCs, NFAT2 was localized in the cytoplasm compared to the untreated PBMCs which shows the nuclear localization of NFAT2. These results indicate that LL-37 blocks the nuclear translocation of NFAT2. Moreover, Supanchart et al. also reported that LL-37 treatment significantly inhibits osteoclastogenesis by blocking TLR9 signaling, whereas the co-incubation of LL-37 with CpG ODN (TLR9 agonist) reverses the inhibitory effect of LL-37 on osteoclast differentiation [103]. Consistently, Horibo et al. found that LL-37 suppresses osteoclastogenesis in human PBMC cultures but not in mouse bone marrow macrophages (BMM) cultures [104]. They found that cathelicidin-related antimicrobial peptide (CRAMP) has no effect on RANKL-induced osteoclastogenesis in BMM cultures even though BMM highly expresses a receptor of CRAMP-formyl peptide receptor 2. Additionally, they found that the failure effect of CRAMP on osteoclast differentiation and proliferation in BMMs induced by RANKL is likely due to its modest effect on ERK (extracellular signal-regulated kinase) phosphorylation in BMMs. Moreover, CRAMP inhibits RANKL expression in osteoblasts treated with LPS (TLR4 ligand) and flagellin (TLR5 ligand) but not in osteoblasts treated with RANKL. Their results further demonstrated that the positively charged fragment of CRAMP binds to the negatively charged fragment of LPS and flagellin, resulting in the inhibition of the interaction of LPS and flagellin with TLRs. Thus, this neutralization protects bone resorption induced by bacterial infection [104].

**Figure 2:**
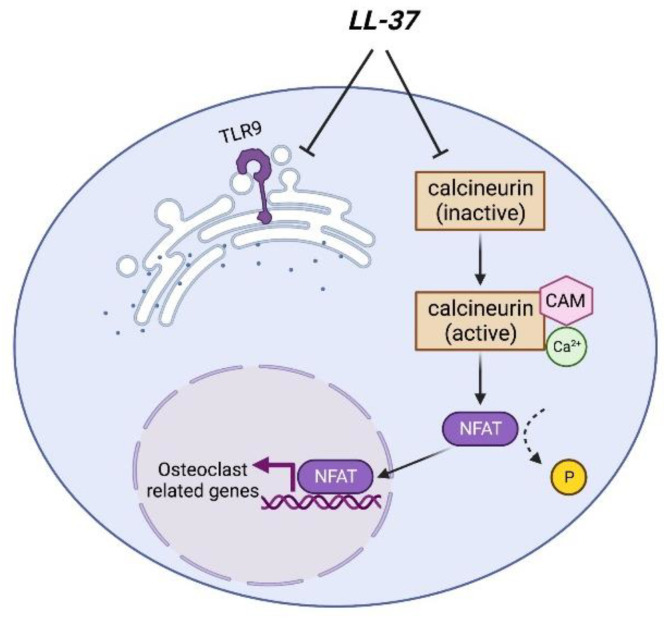
Inhibition of osteoclastogenesis via LL-37. LL-37 blocks TLR9 signaling during osteoclast formation. Ca^2+^/calmodulin (CAM) activates calcineurin, a calcium and calmodulin-dependent serine/threonine protein phosphatase. Activated calcineurin dephosphorylates NFAT protein which leads to NFAT nuclear translocation and the induction of osteoclast-related gene expression. LL-37 inactivates calcineurin activity, thus preventing the nuclear translocation of NFAT2.

P2X_7_ receptor, ATP release, and adenosine signaling in human have been reported to be essential for osteoclast formation [105]. However, how LL-37 regulates these signaling and osteoclasts remains to be further investigated. Supanchart et al. showed that the antibody against P2X_7_ does not reverse the inhibition of osteoclastogenesis by LL-37 in human PBMC [101]. In Figure 2, we provide a starting point for the mechanism involved in the inhibition of LL-37 in osteoclastogenesis in which LL-37 blocks TLR9 signaling and NFAT2 nuclear translocation. However, the interaction between LL-37 and the receptors of osteoclasts needs to be investigated.

It is worth mentioning that LL-37 has been reported to convert monocytes to a special cell type capable of mineralization, named monoosteophils. Monoosteophils are a new type of bone-forming cell with different cell surface markers compared to MSCs. Hence, the microenvironment can influence the ultimate phenotype of monocytes. For example, in the bone marrow, M-CSF and RANKL stimulate monocytes to differentiate into osteoclasts, but in a wound-healing environment, LL-37 induces monocytes to become monoosteophils [106]. When human monoosteophils were implanted in a freshly drilled hole in mid-diaphyseal femurs of mice, a dramatic amount of bone formation was achieved [107].

### 2.2. Osteoblasts

In addition to regulating osteoclastogenesis and bone resorption, LL-37 also regulates osteogenesis and bone formation. Yu et al. found that LL-37 induces bone marrow stromal cells (BMSC) osteogenesis through the stimulation of the P2X_7_ receptor, which then activates the MAPK signaling pathway to trigger an intracellular cascade resulting in the proliferation, differentiation, and migration of BMSCs [108]. Most recently, Shen X et al. prepared nanotube and nanopore coatings (NP) on the titanium (Ti) surface and found that these NPs have great potential to load antibacterial LL37 peptide and sustainedly release LL-37 for 7 days. Additionally, LL37-loaded NP substrates exhibit a significant increase in bactericide and osteoblast differentiation and mineralization in vitro. Meanwhile this system significantly promotes osteogenesis in both uninfected and infected models [109]. Jin M et al. developed a functional molybdenum disulfide (MoS_2_)/polydopamine (PDA)-LL-37 coating on titanium dioxide (TiO_2_) implant and found that this LL-37-coated system can promote the adhesion and proliferation of MSCs as well as osteoblast differentiation and function evidenced by the increase in alkaline phosphatase (ALP) activity, collagen secretion, and ECM mineralization, along with the increased expression of osteoblastic marker genes including runt-related transcription factor 2 (RUNX2), ALP, and osteocalcin [110]. Besides these findings, Cheng Q et al. found that LL-37 can promote odontogenesis and osteogenesis from stem cells of the apical papilla (SCAPs). They found that treatment with 2.5-μg/mL LL-37 upregulates the proliferation and migration of SCAPs, odonto/osteogenic markers’ expression including dentin sialophosphoprotein, dentin matrix protein 1, RUNX2, and osterix, and promotes ALP activity through the activation of the Akt/Wnt/β-catenin signaling pathway [111]. These studies highlight that LL-37 plays important roles in the regulation of both osteoclastogenesis and osteogenesis.

## 3. Role of LL-37 in the Oral Cavity

LL-37 is an important regulator in the maintenance of the physiological microbiota of the oral cavity and in the pathogenesis of oral dysbiosis, microbia-mediated infectious diseases, autoimmune diseases, and oral carcinomas. These studies have been reviewed by Tokajuk J et al. [112]. Here, we will highlight the recent studies about the role of LL-37 in dental caries, dental pulp, and periodontium.

### 3.1. Dental Caries

Dental caries is an infectious and multifactorial disease affecting a significant proportion of the world’s population. Dental caries is influenced by host and dietary factors besides bacterial infection [113,114]. *Streptococcus mutan (S. mutans)* is the main bacteria involved in dental caries. One of the well-known virulence factors of *S. mutans* is its ability to produce cariogenic biofilms adhered on teeth surfaces and therefore increase bacterial resistance to most antibiotics and therapeutic agents [115,116].

Cathelicidin peptides are localized in the salivary glands, lingual epithelium, and palatal mucosa in mice, and the submandibular duct in humans [61,62]. There is a discrepancy among the findings whether LL-37 could play a protective role against caries. For example, one study showed that cariogenic species are resistant to LL-37, especially when biofilm formation occurred [117]. A few other studies demonstrated no significant correlation between the level of LL-37 and caries experience in children [118,119,120], whereas another study reported that there is a higher concentration of saliva LL-37 in caries-free children and children with low to moderate caries activity [121]. Moreover, Gua et al. [122] assessed the anti-infective ability of epigallocatechin gallate (EGCG), the most abundant constituent of tea catechins, and LL-37 and found that LL-37 enhances the impact of the antibiofilm effect of EGCG on *S.mutans* by promoting the interaction between EGCG and lipoteichoic acid (LTA) of *S. mutans* [122]. The discrepancy of the above results may be due to several factors such as different racial/ethnic background, different technique (ELISA vs. slot blot), and the collection of stimulated or unstimulated saliva. However, the precise role and mechanism of LL-37 in dental caries need to be further investigated.

### 3.2. Dental Pulp

Lesions of endodontic origin typically develop from exposure of the pulp to oral bacteria because of the tooth integrity disruption resulting from carious lesions that penetrate the tooth, and the iatrogenic causes such as the mechanical exposure and fracture of the tooth. Permanent damage of the dental pulp may occur from the pulpal infection under the exposure site [123,124,125]. Elimination of bacteria by AMPs such as LL-37 can inhibit the inflammation and the migration of pulp cells yielding a favorable environment for the regeneration of pulp–dentin complexes [126]. Kajiya M et al. reported that LL-37 stimulates the migration of human pulp (HP) cells to increase the regeneration of pulp–dentin complexes through the activation of the epidermal growth factor receptor (EGFR) and the c-Jun N-terminal kinase (JNK) signaling pathways [127]. Moreover, LL-37 has been reported to boost the secretion of VEGF from human pulp cells, and then promote angiogenesis and facilitate pulp wound healing [128]. VEGF is expressed not only from endothelial cells but also fibroblasts in the dental pulp [129]. Thus, LL-37 may act on both endothelial cells and pulp cells to promote angiogenesis and accelerate the healing process particularly at the pulp injury site. Recently, LL-37 has been proved to be biocompatible and able to enhance odontoblast proliferation and differentiation from dental pulp stem cells (DPSCs) in a dose-dependent manner as well as increase the expression of the dentin sialophosphoprotein (DSPP), a marker gene of odontoblastic differentiation [130]. In addition to DPSCs, the proliferation and differentiation of stem cells from the apical papilla (SCAPs) at the root apex, essential for the formation and maturation is also regulated by LL-37 through the activation of the Akt/Wnt/b-catenin signaling pathway [111]. These studies highlight the important role of LL-37 in dental pulp repair and regeneration.

### 3.3. Periodontium

The periodontium consists of the periodontal ligament, cementum, gingiva, and alveolar bone and is the supporting structure for the teeth [131]. Periodontitis is a chronic inflammation that leads to loss of teeth through the destruction of the periodontium [132]. Gram-negative anaerobic bacteria mostly colonized at the subgingival sites activate the host immune response and are closely associated with periodontitis [133]. Although the adjunctive use of antibiotics in combination with mechanical debridement facilitates bacterial reduction, the success of the antibiotic strategy is limited due to the development of bacterial resistance [134,135]. Given this, new strategies related to AMPs usage have been studied to combat microbial pathogens.

AMPs have a broad antibacterial activity against pathogens associated with periodontitis [136]. Gingival epithelial cells secrete several AMPs either constantly or in response to an infection [136,137]. AMPs such as LL-37 interact with negatively charged phospholipids at the outer surface of bacterial membrane due to amphipathicity and their positive charge to interfere with the membrane’s integrity leading to pore formation and cell lysis [2]. LL-37 has demonstrated antimicrobial activity against periodontal pathogens such as *Actinobacillus actinomycetemcomitans* (*A.a.*) and *Capnocytophaga* spp. [138]. *A.a.* is one of the key pathogens associated with the rapid destruction of the periodontium, which often affects younger individuals [139]. *Capnocytophaga* spp. is involved in gingivitis, periodontitis, and advanced periodontitis in juvenile diabetics [140]. Sol et al. found that *A.a.* shows resistance to high concentrations of LL-37; however, under low and submicrocidal concentrations, LL-37 can bind to *A.a.* via hydrophilic and hydrophobic interactions and inhibits its biofilm formation. LL-37 efficiently opsonizes *A.a.* and enhances the removal of *A.a.* by neutrophils [141]. *Porphyromonas gingivalis* (*P. gingivalis*), a black-pigmented, Gram-negative, anaerobic coccobacillus, is one of the most important species in the pathogenesis of chronic periodontitis [142]. Walters et al. examined the ability of AMPs to inhibit LPS release from *P. gingivalis* and found that LL-37 is the most potent inhibitor of cytokine production and can completely neutralize *P. ginigivalis* LPS [143]. These observations demonstrate that LL-37 may be a promising drug target for the treatment of periodontitis.

The significance of LL-37 in the prevention of periodontitis is evident in patients with Kostmann syndrome (an autosomal recessive disorder characterized by severe neutropenia) who suffer from severe periodontal disease. Those patients lack LL-37 in their saliva or in their neutrophils compared to healthy individuals. Administration of granulocyte-colony-stimulating factor (G-CSF) increases the number of neutrophils, but patients still lack LL-37 and manifest periodontal diseases. However, transplantation of bone marrow into those patients can restore both the number of neutrophils and the level of LL-37 to the normal level, which leads to the significant inhibition of periodontal diseases [144,145]. Likewise, patients with Papillon–Lefevre syndrome and Haim–Munk syndrome develop periodontitis due to the low levels of LL-37 caused by lost-of-function mutations in the cathepsin C (CTSC) gene. As a result, neutrophils lack serine protease activity, which is essential for LL-37 production from its precursor. These patients have a normal amount of LL-37 precursor—hCAP18—but little is processed to the active LL-37 allowing for infection with *A.a.* and the development of severe periodontal disease [146,147]. Addition of synthetic LL-37 augments host LL-37 levels which may contribute to periodontal disease resolution. McCrudden et al. revealed that synthetic/exogenous LL-37 is stable in healthy GCF (gingival crevicular fluid); however, it rapidly degrades by GCF from periodontitis patients [148]. It has been shown that Tannerella forsythia and *P. gingivalis*—which are parts of the red complex—are capable of suppressing LL-37 function [149,150]. Additionally, Tanaka et al. revealed that serum and NaCl reduce the antimicrobicidal activity of LL-37. Thus, a membrane-delimited condition such as liposome, which excludes exogenous factors such as salt and serum proteins may optimize the antimicrobial activity of LL-37 [138].

Besides the antimicrobial activity, LL-37 modulates the host immune response [151]. LL-37 induces interleukin-8 production from myeloid and epithelial cells, which is required for neutrophil infiltration into the infectious site [152]. Treatment of periodontal ligament (PDL) cells with LL-37 has been shown to prevent the LPS-induced stimulation of MCP-1 (monocyte chemoattractant protein-1) expression. Pre-exposure of PDL cells with LL-37 followed by stimulation with LPS prevents LPS-induced MCP-1 upregulation in the absence of LL-37, demonstrating that LL-37 functions intracellularly rather than neutralizing LPS. Mechanistically, LL-37 seems to be independent of the interaction with with NF-κB (Nuclear Factor Kappa B) transcriptional factor and may directly interact with pro-inflammatory MCP-1 gene to induce transcriptional and/or post-transcriptional effects [153]. Additionally, the combination of LL-37 and human Beta-Defensin-3 synergistically reduced the secretion of cytokines by *A.a.* LPS stimulation in a 3D co-culture model of gingival epithelial cells and fibroblasts [154]. These findings indicate that LL-37 plays a crucial role in the innate immunity of periodontal tissue.

It is necessary to design a stable synthetic of LL-37 to combat oral pathogens as a therapeutic remedy to periodontitis. An amidated form of LL-37, such as pentamide-37, expressed almost the same anti-microbial activity and less sensitivity to serum inhibition [138]. Perhaps the pentamide-37 can be considered a more suitable alternative to LL-37 for therapeutic application in the presence of serum. LL-37 and its modified forms may have promising anti-inflammatory potential as an adjunctive therapy for the treatment of periodontitis.

## 4. Regeneration

The successful rehabilitation of oral functions in patients with inflammation-induced bone loss such as periodontitis and rheumatoid arthritis, the edentulous atrophic alveolar process, or the reconstruction of large bone defects caused by trauma or tumor, are complex and challenging [155,156,157]. Some methods for bone and periodontium regeneration such as autograft (tissue from one part to another part of the same body) and allograft (tissue from the same species, i.e., humans) have drawbacks and limitations including the need for second surgery, limited availability, donor site morbidity, disease transmission, and immunogenicity and rejection in case of autograft and allograft [155,158]. Bone tissue engineering has offered promising alternatives to grafts via using growth factors, scaffolds, and stem cells to promote bone regeneration [159]. LL-37 has been explored as a potential compound with bone and periodontium regeneration, which are summarized in the below sections.

### 4.1. Role of LL-37 in Bone Regeneration

To promote bone regeneration, the proliferation and osteogenic differentiation of progenitor cells are needed and angiogenesis is also essential, because the newly formed vessel can deliver oxygen, nutrition, and stem cells to the core of bone defects [160,161]. Studies have shown that LL-37 promotes angiogenesis and/or osteogenesis during bone regeneration. Kittaka et al. first reported that LL37 induces new bone formation in a rat calvarial defect model. They found that more STRO-1 (a MSCs marker) positive fibroblastic cells and CD34 positive endothelial cells accumulated in the bone defect area in the LL-37-treated group compared to the control group without LL-37 treatment. They further found that LL-37 does not affect the osteogenesis gene expression in MSCs and mineralization. Thus, those results demonstrate that LL-37 only contributes to the accumulation of MSCs to the bone defect site, and LL-37 promotes bone regeneration by inducing new blood vessel formation and recruiting MSCs, but not affecting osteogenic differentiation of MSCs [89]. Nonetheless, our previous study showed that LL-37 especially combined with BMP2 promotes MSC proliferation, migration, and differentiation. Additionally, LL-37 inhibits osteoclastogenesis and bacterial infection. We further found that LL-37 combined with BMP2-modified MSCs substantially enhances bone and blood vessel formation in inflammatory calvarial osteolytic defects [97]. The findings demonstrate that LL37 is a therapeutic agent for enhancing bone regeneration and repair. Consistent with our findings, the most recent study reported that the implantation of LL37 Poly (sebac’oyl diglyceride) (PSeD) gel combined with human adipose-derived mesenchyme stem cells (hADSCs) increases the expression of osteogenic mRNA and protein levels and enhances osteogenic differentiation of hADSCs, which significantly accelerates the bone regeneration process in a rat calvarial bone defect model. Moreover, LL37 inhibits the inflammation after implantation and has a good biocompatibility in vivo [162]. Besides using LL-37, Li et al. used KR-12, the smallest fragment of LL-37 to stimulate osteogenesis. They found that KR-12 promotes the osteogenic differentiation from human bone marrow-derived MSCs (hBMMSCs) in a dose-dependent manner. Mechanistically, they found that KR-12 activates the transcription of BMP2 and the SMAD signaling pathway. Both transforming growth factor-β/SMAD inhibitor (LDN-193189 HCL) and BMP2 small interfering RNA (siBMP2) inhibit SMAD phosphorylation and subsequent osteogenic differentiation induced by KR-12 [163].

### 4.2. Role of LL-37 in Periodontium Regeneration

In addition to regulating bone regeneration, LL37 was recently reported to act as a potent angiogenic inducer through promoting VEGF expression in human PDL cells via the activation of the ERK and NF-kB signaling cascades [164]. Since neovascularization is an essential element of tissue regeneration, LL-37 can be considered as a therapeutic agent for periodontal regeneration [89]. However, LL-37 does not induce PDL cells to differentiate into osteoblasts. Moreover, high concentrations of LL-37 reduce PDL cell proliferation through the inhibition of DNA synthesis and the promotion of apoptosis [75], suggesting the concentration of LL-37 may be critical for periodontium regeneration.

One of the treatment modalities for replacing missing teeth is to use titanium-based dental implants. Titanium has been extensively used in dental implants due to its excellent mechanical properties and good biocompatibility [165]; however, its bio-inert surface limits osteointegration. Another limitation of osteointegration is bacterial infection [166,167]. Nano topographic modifications have been proved to improve osseointegration by increasing the implant’s surface and promoting osteoblast migration. However, these modifications do not provide antimicrobial properties. In order to prepare an antibacterial and pro-osteogenesis implant, Shen et al. loaded LL-37 to titania nanopores (NP) structures. The NP structures can substantially load antibacterial LL37 peptides due to the reservoir characteristics of porous materials, which can achieve the sustained release of LL37 within 7 days. Moreover, the LL37-loaded NP system dramatically improves antibacterial and osteogenic induction abilities resulting in enhanced osteogenesis in both infected and uninfected models in vivo [109]. The recent studies regarding the role of LL-37 in bone and periodontium regeneration have been summarized in Table 1.

## 5. Signaling Pathways of LL-37 Regulation

LL-37 regulates immune defense and tissue regeneration. However, the molecular mechanism by which LL-37 regulates remains largely unknown. Here we briefly summarize the signaling pathways of LL-37 regulation including G protein-coupled receptors (GPCRs), receptor tyrosine kinases (RTKs), ligand-gated ion channel (LGIC), and TLRs [168].

N-formyl peptide receptor 2 (FPR2; formerly known as formyl peptide receptor like-1) is one of the GPCR proteins. It is one of the most studied receptors associated with LL-37. FPR2 was first identified as a functional receptor for LL-37 in 2000. Studies have shown that the activation of FPR2 by LL-37 exbibits different functions in different cell types. De Yang et al. found that LL-37 chemoattracts human peripheral blood neutrophils, monocytes, and T Cells to the microbial invasion site and induces calcium mobilization in monocytes through activating FPR2 signaling [84]. Furthermore, LL-37 was reported to suppress neutrophil apoptosis along with ERK1/2 phosphorylation and inhibit caspase 3 activity via the activation of the FPRL1 and P2X_7_ receptor [71]. Different from the above study, Barlow et al. found that LL-37 inhibits neutrophil apoptosis through the activation of FPRL1 and P2X_7_ and subsequent downstream PI-3K signaling but not the ERK1/2 MAPK pathway [69]. Besides these, LL-37 can stimulate the generation of leukotriene B4 (LTB4)—a potent chemoattractant—from neutrophils via FPR2. Furthermore, LL-37 promotes LTB4 release by affecting the activation of p38MAPK and the phosphorylation of cPLA2 [167,169].

A recent study showed that LL-37 affects the angiogenesis and arteriogenesis depending on binding to the Formyl-peptide receptor-like 1 (FPRL1), and then activating downstream events of FPRL1 via an increase in the intracellular Ca^2+^ level and translocating NF-κB into the nucleus. Interestingly, the MEK inhibitor, PD098059, can prevent LL-37-induced endothelial proliferation, suggesting that ERKs may be involved in LL-37 effects [14]. Consistently, additional evidence has shown that in human fibroblasts, LL-37 induces NADPH oxidase activation via the activation of FPRL1 and the downstream signaling which involves the phosphorylation of ERKs and PKC (protein kinase C) -α and PKC-δ activation [170].

Another receptor associated with LL-37 is RTKs. Tjabringa et al. found that LL- 37-mediated EGFR transactivation in airway epithelial cells via the metalloproteinase-dependent processing of EGFR ligands, and thereby activating the MAPK/ERK signaling pathway resulting in ian ncreased release of IL-8 [171]. Insulin-Like Growth Factor 1 Receptor (IGF1R)—another member of the RTK family—can also be activated by LL-37 which is involved in signaling pathways related to tumor development. The phosphorylation of IGF1R resulted in the activation of the MAPK/ERK pathway but did not affect the PI3K/Akt signaling pathway [172].

**Figure 3:**
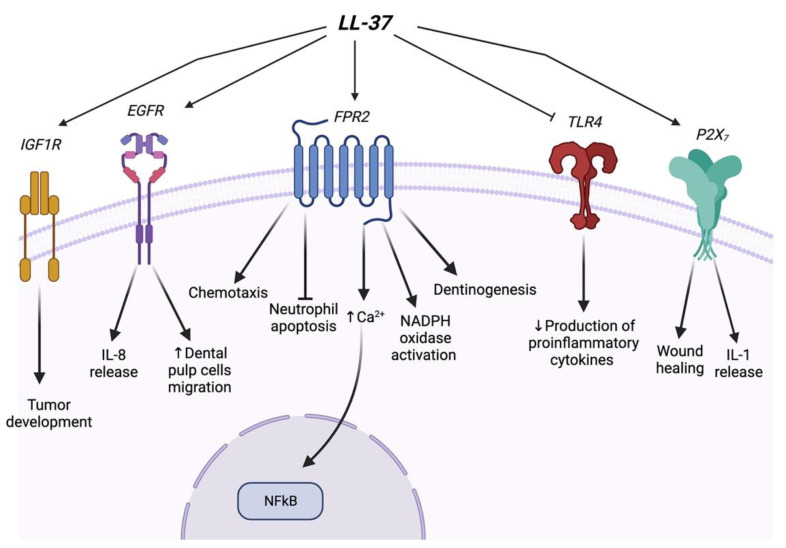
A schematic of the receptors associated with the LL-37 signaling pathway.

In addition to the aforementioned receptors, LL-37 also regulates the TLR family. LL-37 diminishes the production of proinflammatory cytokines by inhibiting TLR4 in monocytes and macrophages, which is valuable for limiting the induction of systemic inflammatory syndrome/septic shock [173]. Moreover, the overexpression of LL-37 induces the development of autoimmunity. For example, extracellular self-RNA fails to reach endosomal compartments of dendritic cell (DCs) due to the rapid degradation by RNases. Yet LL-37 can form a complex with extracellular self-RNA which is highly protected from RNase activity. As a result, self-RNA accesses the endosomal compartments of DCs and stimulates the TLR7 and TLR8 signaling pathways [174]. LL-37 also forms a complex with self-DNA and TLR9 signaling activation in DCs [174]. The proinflammatory response of LL37 such as IL-1 secretion has been reported via the direct activation of P2X_7_ receptors leading to a transient release of ATP, membrane permeability, and activation of caspase-1 [175]. KN-62 (P2X_7_ antagonist) suppresses fibroblast proliferation and growth induced by LL-37, which demonstrates the impact of LL-37 in the regulation of the P2X_7_ pathway. Fibroblasts play a crucial role in wound healing by synthesizing the extracellular matrix [92], indicating that LL-37 may play an important role in wound healing and tissue repair. LL-37 plays a crucial role in periodontal immunity by modulating the host immune response besides its antimicrobial and antibiofilm activities [120,149,150]. Studies have shown that LL-37 stimulates IL-8 secretion which is the primary mediator of innate host defense [150]. Additionally, LL-37 inhibits the expression of LPS-induced stimulation of MCP-1 and decreases the production of IL-6 [75,149]. No studies have yet investigated the receptors associated with LL-37 function in periodontal health and diseases. However, one study found that CRAMP binds to FPR2 in rat odontoblasts to induce physiological and reparative dentin formation [176]. In addition, LL-37 activates the EGFR and JNK signaling pathway to promote dental pulp cells migration which is a key step in pulp–dentin complex regeneration [127]. Even though multiple functions of LL-37 have been mediated by a variety of receptors and transmembrane channels, it is still elusive how one small peptide with low structural complexity can activate independent receptors. To deeply understand the mechanism, Verjans et al. conducted docking experiments with the established nuclear magnetic resonance (NMR) structure of LL-37 and revealed the essential domains of receptor involved in LL-37 binding [168]. These findings have increased the knowledge of the underlying molecular mechanisms in LL- 37-induced receptor activation. A summary of the receptors associated with LL-37 and downstream function is provided in Figure 3.

## 6. Summary

LL-37—the sole human cathelicidin-derived antimicrobial peptide—has diverse functions in the regulation of wound healing, chemotactic activity, release of inflammatory mediators, cell differentiation, and apoptosis. In addition, LL-37 was proposed to act as a transcription factor by binding to nuclear DNA in a specific manner which elucidates another function of this small peptide [177]. Currently, a new appealing picture of LL-37 has arisen in regeneration, as reviewed in this paper. In conditions such as bone fractures, periodontitis, apical periodontitis, and osteomyelitis, there is a high risk of bacterial infection. Therefore, promising bone regenerative agents should also possess an anti-microbial effect to prevent infection. LL-37 may open a new horizon in bone and periodontium regeneration in addition to its broad-spectrum antimicrobial activity. Despite the advantages of LL-37, there are several limitations and drawbacks that should be taken into consideration including susceptibility to protease degradation, high cost to synthesize, and toxicity to human cells at high concentrations [178,179]. Although some specific molecular pathways have been proposed for the diverse functions of LL-37, it is still poorly understood. Future studies should assess the molecular pathways associated with LL-37, especially in the regeneration process.

## Figures and Tables

**Table 1:** Summary of the studies regarding the role of LL-37 in bone and periodontium regeneration.

Authors	Results	Reference Number
Kittaka et al. (2013)	LL37 enhances angiogenesis and recruits stem cells to promote bone regeneration in rat calvarial defects.	[87]
Liu et al. (2018)	LL37 increases the migration, proliferation, and osteogenic differentiation of MSCs in addition to inhibiting osteoclast differentiation. LL-37 combined with BMP2 dramatically promotes MSCs- mediated angiogenesis and bone regeneration in LPS-induced mouse calvarial osteolytic bone defects.	[95]
Li et al. (2021)	Application of LL-37 accompanied with PSeD gel and hADSCs significantly accelerates the process of bone regeneration through enhancing osteogenic differentiation and reducing inflammation in rat calvarial bone defect.	[160]
Li et al. (2018)	KR-12 (the smallest fragment of LL-37) stimulates the osteogenic differentiation of hBMMSCs via the activation of the BMP/SMAD pathway signaling pathway	[161]
Kittaka et al. (2013)	LL-37 contributes to periodontal regeneration through upregulating VEGF-A expression resulting in the activation of ERK and NF-kB signaling cascades in HPL cells and inducing angiogenesis.	[162]
Shen et al. (2019)	LL-37-loaded NP structure significantly improves cell adhesion, spreading, and osteogenic differentiation. Implantation of LL37-loaded NP structures into infected and uninfected rat femurs significantly improves bone formation.	[107]
